# Sex Differences in Progression of Diabetic Cardiomyopathy in OVE26 Type 1 Diabetic Mice

**DOI:** 10.1155/2020/6961348

**Published:** 2020-05-14

**Authors:** Xiaoqiang Tang, Saizhi Jiang, Jian Zhang, Shanshan Zhou, Yang Zheng

**Affiliations:** ^1^The Center of Cardiovascular Diseases, The First Hospital of Jilin University, Changchun 130021, China; ^2^Pediatric Department, The First Affiliated Hospital of Wenzhou Medical University, Wenzhou 325000, China

## Abstract

OVE26 mice are a widely used transgenic model of early-onset type 1 diabetes. These mice overexpress calmodulin in their pancreatic *β* cells, develop severe diabetes within the first weeks of life, and progress to severe diabetic complications including diabetic nephropathy and diabetic cardiomyopathy (DCM). To date, diabetic nephropathy in OVE26 mice has been well explored, leaving the progression of DCM and the gender impact in this type 1 diabetes model still unrevealed. In our study, male and female OVE26 mice and age-matched nondiabetic FVB mice were examined at 4, 12, 24, and 36 weeks for their cardiac function, body weight, blood glucose, and heart weight/tibia length ratio. Further, histopathological examination and Western blot analysis for the key markers demonstrate that DCM appears at 24 weeks OVE26 mice, initiating with cardiac senescence, followed by fibrosis and then cardiac dysfunction. Mitochondrial respiration function analysis showed no indication of dysfunction in OVE26 mice at 24 weeks of age in both genders. In addition, no significant difference for the pathogenic progression was observed between OVE26 and FVB mice in both males and females. In conclusion, this study suggests cardiac senescence and fibrosis, which may be amended by sex differences, play key roles in the progression of DCM in OVE26 mice. The comprehensive characterization of diabetic cardiomyopathy progression and the sex difference impact in OVE26 mice provides a basis for future study on DCM using OVE26 mice.

## 1. Introduction

Diabetic cardiomyopathy (DCM) is defined as myocardial dysfunction due to abnormal myocardial structure and reduced contractility in the absence of noticeable vascular complications in patients with diabetes mellitus [1]. It was first observed in the 1970s when four patients exhibited concomitant diabetes and heart failure without the well-recognized casual factors such as coronary artery disease (CAD), hypertension, and significant valvular disease [2]. In the follow-up studies, DCM is typically characterized by fibrosis and hypertrophy, and eventually cardiac dysfunction [3]. In fact, diabetes, independent of CAD and hypertension, increase the incidence of heart failure by 2.5- to 5-fold in the Framingham Heart Study [4]. The reality that diabetic cardiomyopathy appears in both type 2 diabetes (T2D) and type 1 diabetes (T1D) implicates that it is likely the direct pathological effects of diabetes on the myocardium, rather than the etiology, that plays a causal role in the development of DCM.

OVE26 mouse is a transgenic model that overexpresses calmodulin in pancreatic *β* cells, which would result in a deficiency in the production and secretion of insulin (T1D) due to the *β* cells damage [1]. Owing to the well-characterized cardiac and renal complications, the OVE26 mouse model is frequently used to study complications caused by diabetes [5, 6].

To date, several studies on cardiomyopathy using OVE26 mice have shown T1D is directly associated with alterations in cardiac structure and cardiac dysfunction in these mice [7–9]. However, most of these studies used mice age from 4 to 18 weeks, which cannot reflect the dynamic progression of DCM in the older mice. Another problem is that the male mice are mainly used for these studies; therefore, the effects of sex differences on the complications are largely ignored. Given that the female mice are significantly distinct from the male mice in the development of diabetic nephropathy in the OVE26 model [10, 11], there is a necessity to also consider the effects of sex on the progression of DCM in OVE26 model.

The aim of this study was thus to dissect the development and progression of the features of cardiomyopathy in OVE26 mice and to determine the impact of sex and age in these processes. These together would greatly facilitate the utilization of the OVE26 model in the research of DCM.

## 2. Materials and Methods

### 2.1. Animals

OVE26 mice on the FVB background were maintained in the Research Resources Center at the University of Louisville as described previously. All mice were given free access to food (standard chow diet) and water without insulin. All animal procedures conformed to the Guide for the Care and Use of Laboratory Animals by NIH and the Jilin University Animal Care and Use Committee.

The mice were sacrificed at 4-, 12-, 24-,30-, and 36-week-old (*N* = 5 − 8), respectively, and spot urine was collected one day before sacrifice. Body weight was measured before the mice were anesthetized with Avertin. Whole blood was collected from the inferior vena cava with a lithium heparin tube (BD, Franklin Lakes, NJ, USA). After centrifugation (4000 rpm, 20 min, 4°C), plasma was transferred to 1.5 ml Eppendorf tubes and stored at -80°C. Hearts were collected for weight measurement. The right tibia was collected and measured for the length.

### 2.2. Echocardiography

To assess the heart function of the mice, transthoracic echocardiograms were performed using a Visual Sonics Vevo 770 high-resolution imaging system, as described before [12]. Briefly, mice were anesthetized by intraperitoneal (IP) injection of Avertin (240 mg/kg) and placed in a supine position on a heated platform to maintain body temperature. Two-dimensional and M-mode echocardiography was used to assess wall motion, chamber dimensions, and cardiac function.

### 2.3. Sirius Red Staining

After anesthesia, the mouse hearts were collected and fixed in 10% formalin solution, then dehydrated in graded series of alcohol, removed with xylene, embedded in paraffin, and cut to a thickness of 5 *μ*m. Tissue sections were deparaffinized and rehydrated for Sirius red staining of collagen using 0.1% Sirius red F3BA and 0.25% fast green FCF [13]. Collagen-containing tissues were stained red, while myocardial tissues were stained green. ImageJ was used to estimate collagen content (red area).

### 2.4. Western Blot for Protein Expression

The heart tissue from each animal was snap-frozen and stored at -80°C. The tissue sample was homogenized and suspended in lysis buffer (with 1 mM NaF, 1 mM Na_3_VO_4_, and before use 1 mM PMSF and 1/200 protease inhibitor mixture) at 4°C for 4 hours. The lysate was centrifuged (12,000 g, 4°C, 20 min), and the supernatant was collected. Protein concentration was determined using a Bradford protein-binding assay. Total protein was separated on an SDS-PAGE (8%, 10%, or 12% acrylamide) gel and transferred to a nitrocellulose membrane. After blocking at room temperature for 1 h in 5% milk/TBST, the membrane was incubated at 4°C overnight with anti-*β*-actin antibody (1 : 5000, Santa Cruz, SC-47778); antifibronectin antibody (1 : 1000, Abcam, ab2413); or anticollagen I antibody (1  : 1000, Abcam, ab34710); 3-nt antibody (1 : 1000, Millipore, ab5411) and 4-hne antibody (1 : 1000, Alpha Diagnostic Int, HNE11-S); P53 (1 : 1000, cell signaling, #2524), P21 (1 : 1000, Santa Cruz, SC-6246), P16 (1 : 1000, Abcam, ab189034), MMP12 antibody (1 : 1000, Abcam, ab52897); Il-1*β* (1 : 1000, Abcam, ab9722), VCAM (1 : 1000, Abcam, ab134047), ICAM (1 : 1000, Abcam, ab179707). The membrane was washed three times for 5 min in PBST and incubated with secondary antibody for 1 h at room temperature. The signal was detected with enhanced chemiluminescence detection reagents. The relative density of protein bands was quantified using the Image lab 5.2 software (Bio-Rad, USA).

### 2.5. Statistical Analysis

The results are presented as the means ± standard deviations (SDs). Significance was determined by Student's *t* test, Welch's *t* test, one-way ANOVA, and two-way ANOVA. Comparisons with *P* values <0.05 were considered statistically significant.

## 3. Results

### 3.1. General Features of Wide-Type FVB and OVE26 Mice

**Figure 1:**
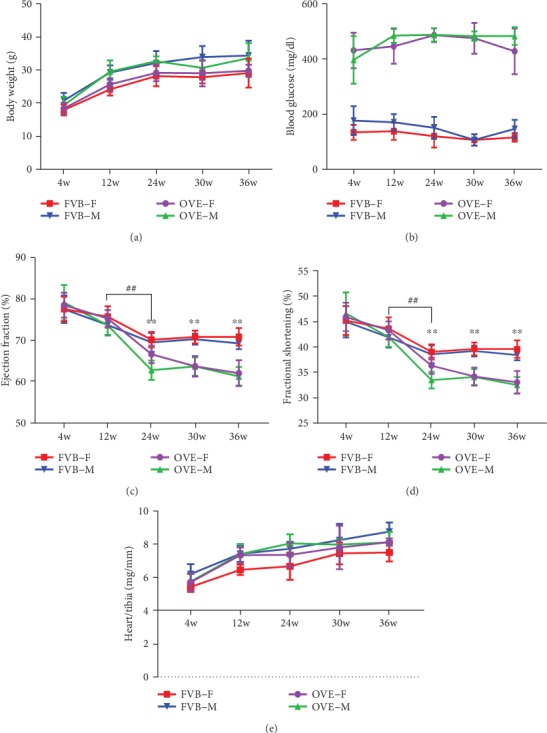
Physical and metabolic characteristics in OVE26 and nondiabetic FVB mice and cardiac function. (a) The body weight of OVE26 mice and FVB mice. (b) The blood glucose in OVE26 mice and FVB mice (*n* ≥ 5). Also, the OVE26 and FVB EF (c) and FS (d). (e) The heart/tibia value in OVE26 and FVB mice. Data are presented as mean ± SEM. ∗∗ vs. sex- and age-matched FVB, *P* < 0.01; ^##^24w sex- and genotype-matched vs. 12w, *P* < 0.01.

The impacts of sex on body weight in both FVB and OVE26 mice at different ages are shown in Figure 1(a). In general, body weight increased over time, with the body weights of the males slightly higher than females in both genotypes and at all ages. There is no significant difference between FVB and OVE26 mice in terms of body weights.

In FVB mice, blood glucose was stable during aging and similar in males and females (Figure 1(b)). In comparison, both male and female OVE26 mice developed hyperglycemia at 4-week of age, and the hyperglycemia continued throughout their lifespan in the range of 370–450 mg/dl (nonfasted blood glucose).

### 3.2. Cardiac Dysfunction in OVE26 Mice

Mouse cardiac functions were examined with echocardiography, and the results were detailed in Table 1. In summary, interventricular septum (IVS), left ventricular internal systolic diameter (LVID), left ventricular posterior wall (LVPW), left ventricular systolic volume (LV Vol), and left ventricular mass (LV Mass) were not significantly changed during aging in FVB mice. Interestingly, female FVB mice had lower LVID, LV Vol, and LV Mass compared with the males at 36 weeks. In both female and male mice, ejection fraction (EF, Figure 1(c)) and fractional shortening (FS, Figure 1(d)) gradually decreased with aging (Table 1). However, OVE26 mice had a greater decrease over time compared to FVB, by the age of 24 weeks, both genders of OVE26 mice showed significantly lower EF and FS than age- and sex-matched FVB mice. Compared with the female OVE26 mice, the male OVE26 mice had a transient lower EF and FS at 24 weeks (Figures 1(c) and 1(d)). In addition, the LVID of both female and male OVE26 mice was higher than sex-matched FVB mice after 24 weeks (Table 1). These changes indicate T1D is associated with changes in cardiac structure and cardiac dysfunction in OVE26 mice. In terms of hypertrophy, we found that while the heart weight/tibia length ratio of all four groups increased with age, the female FVB mice group increased at a much slower rate (Figure 1(e)), reminding us that the sex difference of background strains (FVB) may interfere with our conclusion in the impact of sex difference in transgenic strains (OVE26).

### 3.3. Pathological Changes for Cardiac Remodeling in OVE26 Heart and the Expression of Proteins Related to Heart Fibrosis in Heart of OVE26 Mice

**Figure 2:**
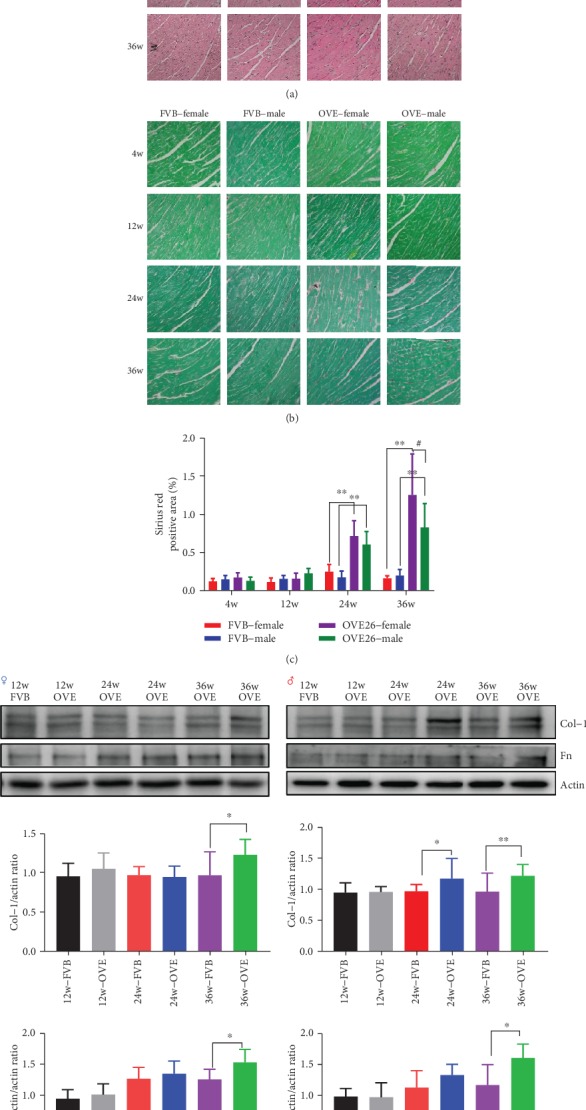
The Sirius red staining of heart tissue in FVB female/male and OVE26 female/male mice and the expression of proteins related to heart fibrosis in heart of OVE26 mice. ^∗^ vs. sex- and age-matched FVB, *P* < 0.05; ^∗∗^ vs. sex- and age-matched FVB, *P* < 0.01; # vs. 36w genotype-matched male, *P* < 0.05.

For both FVB and OVE26 mice, even though the myocardial fibers were more compact and uniform in their younger stages as shown by H&E staining, no significant changes of structural profiles were observed as the mice age increased (Figure 2(a)). However, as an index of fibrosis, Sirius red staining showed that the hearts of OVE26 mice at the age of 24 weeks or older exhibited collagen accumulation (red staining) in both genders, an observation that was absent in the same age FVB mice (Figures 2(b) and 2(c)). Especially, red staining in female OVE26 mice was significantly more extensive than in OVE26 males by 36 weeks. And these data indicate cardiac fibrosis, or at least its initiation, in the OVE26 T1D mice after 24 weeks. To further validate, we examined collagen 1 (Col-1, the expression of which would elevate during fibrosis) and fibronectin (FN, which binds a large number of growth factors that may promote myofibroblast differentiation) by Western blot (Figure 2(d)). Not surprisingly, there was no age-dependent increase of Col-1 in either female or male FVB mice, while the Col-1 accumulation was evident in the diabetic OVE26 mice (36 weeks for females and 24 and 36 weeks for males). There was also a trend increase in FN accumulation in both female and male FVB mice from the age of 24 weeks and then plateaued. Compared with FVB mice, the FN accumulation in OVE26 mice continued to increase after 24 weeks and was significantly higher than their FVB counterparts at 36 weeks for both females and males.

### 3.4. The Expression of Proteins Related to the Oxidative Stress and Damage in OVE26 Mice Heart

**Figure 3:**
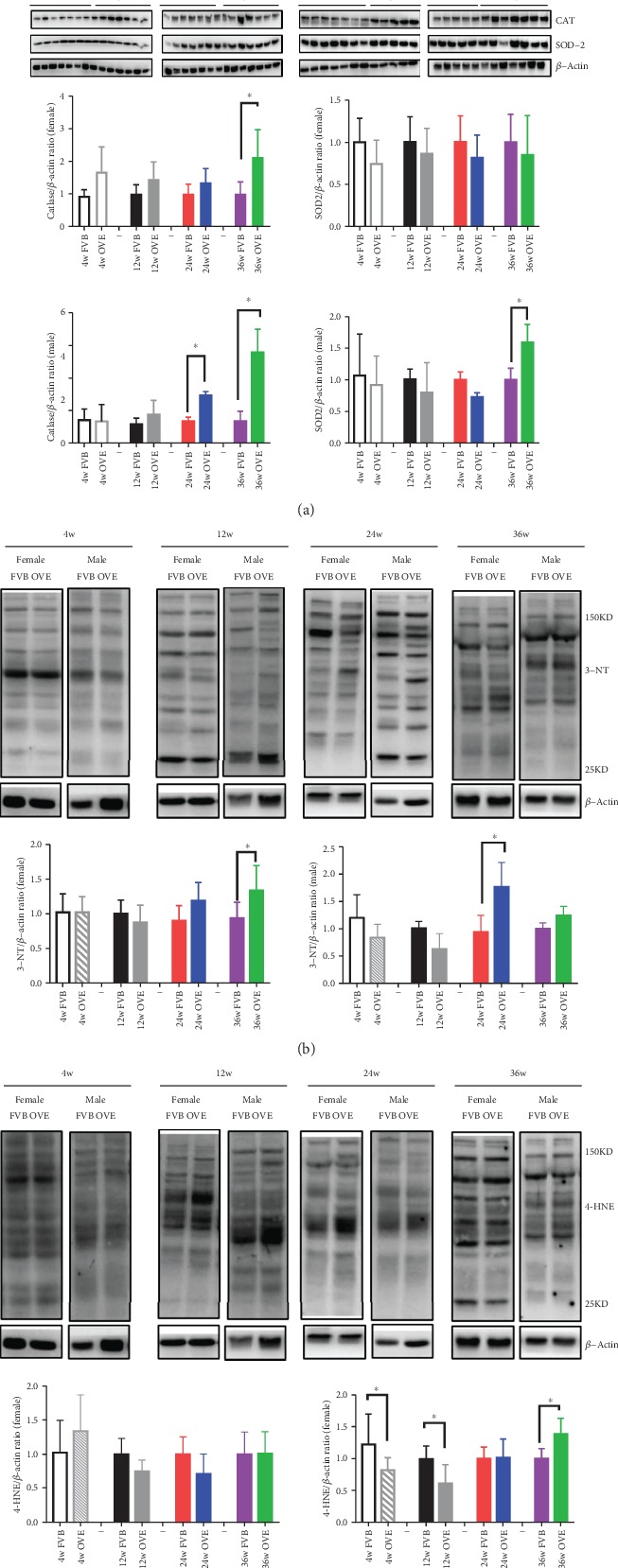
The expression of proteins related to oxidative stress and damage in heart of OVE26 mice. (a) Western blot of the protein expression of CAT, SOD2, and *β*-actin in female and male FVB/OVE26 mice. (b) Western blot of the protein expression of 3NT in female and male FVB/OVE26 mice. (c) Western blot of the protein expression of 4HNE in female and male FVB/OVE26 mice. *N* ≥ 5 in each group ∗ vs. sex- and age-matched FVB, *P* < 0.05.

The elevated fibrosis in the OVE26 heart may be related to diabetes-induced oxidative stress. The commonly used biomarkers of oxidative stress include those in the early response antioxidant system (CAT and SOD2) and the molecules that are consequently modified by interactions with ROS (3-NT and 4-HNE) [14]. While CAT expression stays unchanged within the experiment time course in FVB mice heart for both genders, it was significantly higher in the diabetic OVE26 mice (36 weeks for females and 24/36 weeks for males). These results indicate that the expression of CAT in female mice heart is less sensitive to T1D progression considering that the female OVE26 mice had a much higher basal CAT expression than all other three groups (Figure 3(a)). The cardiac expression of SOD2 also had a similar pattern, although to a less extent (significantly higher only in 36-week male OVE26 mice), ratifying the sex difference in oxidative stress response during the course of diabetes progression. The expression of 3-NT and 4-HNE also followed the same paradigm: 3-NT expression increased in 24-week male OVE26 mice and 36-week female OVE26 mice and 4-HNE expression was only significantly higher in 36-week male OVE26 mice.

### 3.5. The Expression of Proteins Related to the Inflammation in Heart of OVE26 Mice

**Figure 4:**
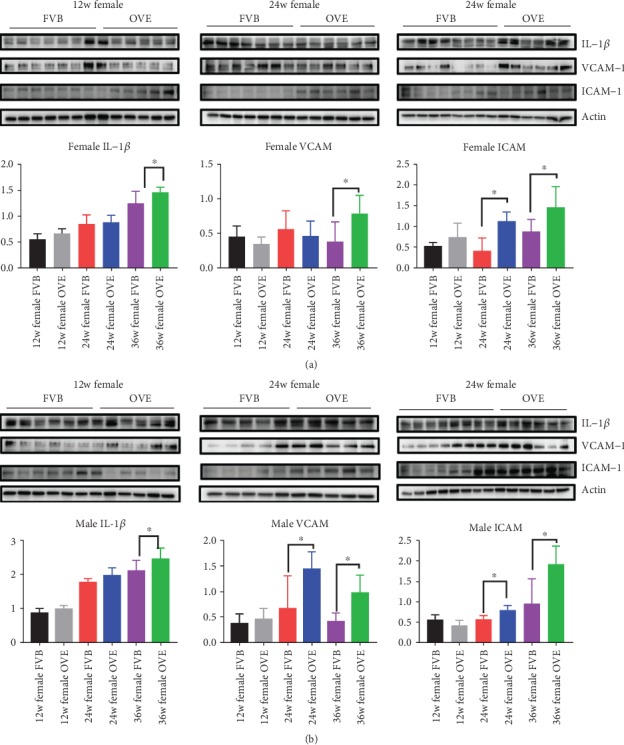
The expression of proteins related inflammation in heart of OVE26 mice. (a) Western blot of the protein expression of Il-1*β*, VCAM, ICAM, and *β*-actin in female FVB/OVE26 mice. (b) Western blot of the protein expression of Il-1*β*, VCAM, ICAM, and *β*-actin in male FVB/OVE26 mice. ∗ vs. sex- and age-matched FVB, *P* < 0.05.

With the discovery of fibrosis and oxidative stress damage in OVE26 mice, we next tested whether T1D caused inflammation in the heart of OVE26 mice. Cause previous research found that the degree of cardiac function damage, myocardial fiber morphology, and expression of oxidative stress markers in mice at 12 weeks and 4 weeks were not much different, so we used 12 weeks mice to compare with 24 and 36 weeks mice. As expected, both FVB mice and OVE26 mice saw expression increase of all three markers of inflammation (Il-1*β*, VCAM, ICAM) (Figure 4). However, both male and female OVE26 mice had significantly higher expression of all markers in the later stage. In detail, Il-1*β* expression was significantly higher at 36 weeks in both male and female OVE26 mice compared to their FVB counterparts; VCAM expression was significantly higher at 36 weeks for female OVE26 mice over FVB females and 24 and 36 weeks for males; ICAM expression was significantly higher at 24 weeks and 36 weeks in both male and female OVE26 mice compared to their FVB counterparts. Again, we found that the males are slightly more responsive in inflammatory factors expression than females following T1D progression.

### 3.6. The Expression of Proteins Related to Heart Senescence and SASP in Heart of OVE26 Mice

**Figure 5:**
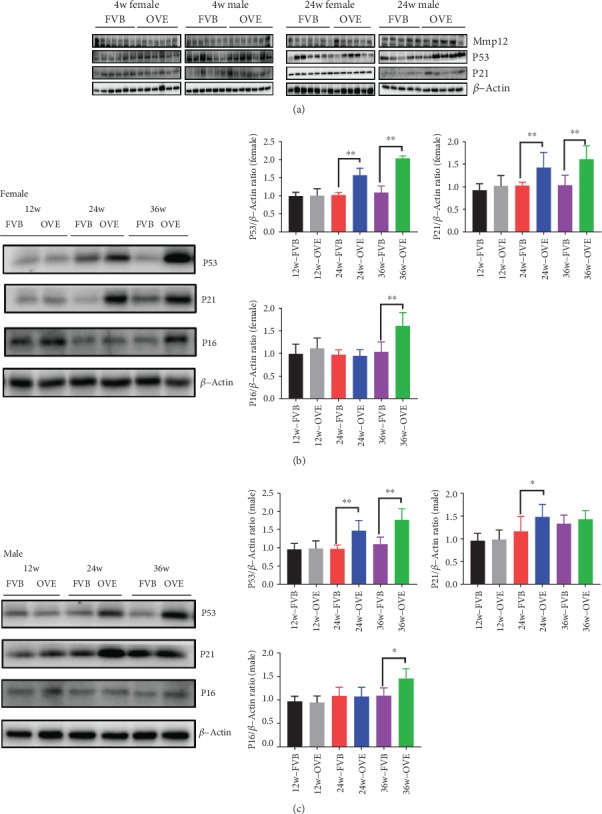
The expression of proteins related to senescence in heart of OVE26 mice. (a) Western blot of the protein expression of MMP12, p53, p21, and *β*-actin in 4 weeks female and male FVB/OVE26 mice. (b) Western blot of the protein expression of MMP12, p53, p21, *β*-actin in 24 weeks female and male FVB/OVE26 mice. (c) Western blot analysis of the protein expression of p53, p21, and p16 at different time points. D. Western blot analysis of the protein expression of p53, p21, and p16 in different time points. *N* ≥ 5 in each group ∗ vs. sex- and age-matched FVB, *P* < 0.05; ∗∗ vs. sex- and age-matched FVB, *P* < 0.01.

The critical role of cellular senescence in cardiac diseases has been recognized [15], and senescence is mainly gauged through the canonical p53/p21 and p16/pRB signaling pathway [16]. Thus, we sought to determine whether senescence happens in OVE26 mice. In FVB mice, the expression of p53, p21, and p16 did not change significantly with age (Figures 5(b) and 5(c)). Interestingly, p53 and p21 were increased in OVE26 mice after 24 weeks in female mice, while p53 also increased from 12 weeks to 36 weeks and p21 increased from 12 weeks to 24 weeks in male mice (Figure 5(b)). In both female and male OVE26 mice, the expression of p16 only increased at 36 weeks (Figure 5(c)). In consistent with a previous report that MMP12 mRNA content significantly increased in the hearts of aging mice [17], we also found the protein levels of MMP12 increased in both FVB and OVE26 mice at 24 weeks, with a higher expression in OVE26 mice, implicating the role of T1D in senescence (Figure 5(a)).

**Figure 6:**
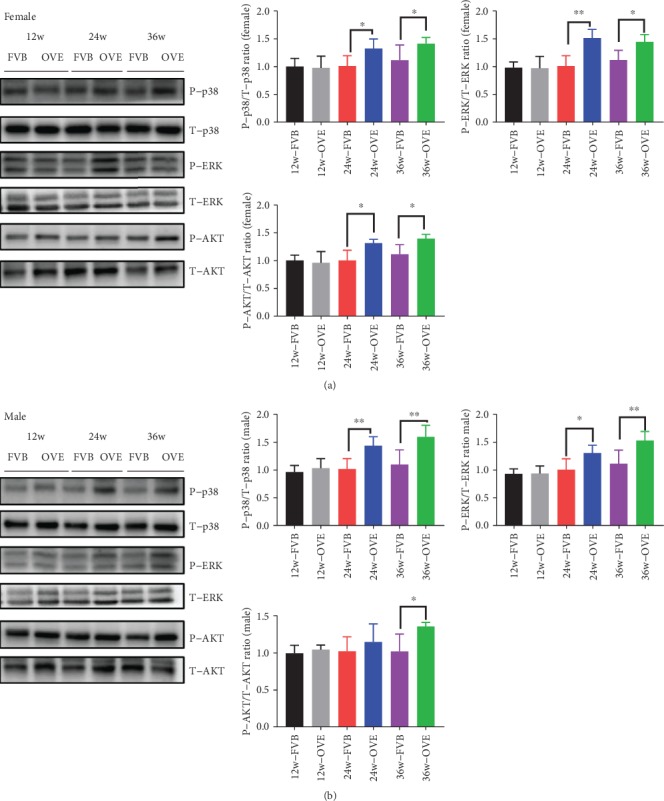
The expression of proteins related to SASP in heart of OVE26 mice. (a) Western blot of the protein expression of P-p38, T-p38, P-ERK, T-ERK, P-AKT, and T-AKT in 12, 24, and 36 weeks of female FVB/OVE26 mice. (b) Western blot of the protein expression of P-p38, T-p38, P-ERK, T-ERK, P-AKT, and T-AKT in 12, 24, and 36 weeks of female FVB/OVE26 mice. *N* ≥ 5 in each group ∗ vs. sex- and age-matched FVB, *P* < 0.05; ∗∗ vs. sex- and age-matched FVB, *P* < 0.01.

During cell senescence, they often display a senescence-associated secretory phenotype (SASP), secreting “SASP factors” such as cytokines, growth factors, and proteases [18]. In addition to the two recognized SASP factors (IL-1*β*, MMP12) we have explored, we further investigated other factors including p38, ERK, and AKT in OVE26 mice. In female and male OVE26 mice, p38 and p-ERK increased significantly at both 12-24 weeks and 24-36 weeks. For p-AKT, the significant increase appeared from 24 to 36 weeks (Figure 6) in female OVE26 mice, whereas in male mice, the significant phosphorylation increase was only seen at 36 weeks (Figure 6).

## 4. Discussion

In the current study, we used the OVE26 mouse model to study the development of DCM in the T1D model. In previous experiments I participated in, we investigated the value of OVE26 mice in understanding the effect of gender on DN [19] but did not mention the effect on the heart. To date, this is the first report of cardiac function in young and adult male and female OVE26 mice. We observed an increase in left ventricular hypertrophy, fibrosis, and diastolic dysfunction in both female and male OVE26 mice compared to the background strain FVB mice. Also, the fibrosis of female OVE26 mice at 36 weeks is more serious than male mice, which is consistent with Sarah D de Ferranti's statistics, which found that women with type 1 diabetes have a greater risk of CVD [20]. Research by Lum-Naihe found that reduced neurofibrin-1 and capillaries in the heart of female diabetic mice may lead to increased damage to the myocardial structure [21]. However, how T1DM modulates this effect in the heart tissues of women versus men is currently unclear. Fibrosis is an important cardiac remodeling that directly leads to the development of DCM [22]. Fibrosis at its early stage is thought to be an adaptive responsive, characterized by the accumulation of excess collagen, which in turn would alter heart structure and cause heart dysfunction. We observed a significant increase in myocardial collagen deposition in OVE26 diabetic mice. The mechanisms by which diabetes induces the cardiac remodeling and cardiac dysfunction include increased collagen deposition with increased expression of transforming growth factor beta (TGF-*β*) and connective tissue growth factor (CTGF), transcription factors that drive collagen production, and in some cases increased activation of poly (ADP-ribose) polymerase 1 (PARP-1) [23]. However, aging-associated cardiac fibrosis is characterized by degenerative changes, such as progressive loss of muscle cells due to necrosis and apoptotic cell death, along with the excessive proliferation of cardiac cells and deposition of ECM [24, 25]. In this process, senescent cells adopt a secretory phenotype (the SASP), which comprises a large number of proinflammatory cytokines, chemokines, and proteases. SASP mediates many of the extracellular functions of senescent cells, such as the secretion of TGF-*β*, which can spread the aging phenotype to peripheral cells in a paracrine manner [26].

Excessive reactive oxygen species (ROS) and superoxides caused by oxidative stress and low-grade inflammation associated with aging underpins age-related cardiovascular dysfunction, namely, left ventricular hypertrophy, fibrosis, diastolic dysfunction, endothelial dysfunction, reduced elasticity of blood vessels, and increase blood vessel hardness [27]. Increased oxidative stress in aging myocardium has multiple consequences, such as increased protein oxidation/nitrification, decreased bioavailability, lipofuscin formation, inflammatory response activation, antioxidative stress response, apoptosis, and endoplasmic reticulum (ER) stress [28]. Our study shows that OVE26 mice had more oxidative stress damage after 24 weeks. Oxidative stress may result from increased ROS generation, a defective antioxidant defense system, or both [27]. Inflammation may be the biological basis of fragile pathophysiology. These changes affect fragility and cognitive decline, as well as the onset of heart disease, neuropathy, and vascular events. If the content of inflammatory compounds exceeds the control of anti-inflammatory compounds, imbalances will occur and an inflammatory state will be established [29]. The key proteins expressed by endothelial cells that bind to inflammatory cells are cell adhesion molecules, VCAM-1 and ICAM-1. ICAM-1 and/or VCAM-1 have a good correlation with cardiovascular disease in patients with diabetes. Becker and colleagues found that patients with type 2 diabetes showed higher levels of sICAM-1, which were not related to known cardiovascular risk and predicted all causes mortality from cardiovascular disease within 10 years [30]. Research by Jager found that elevated sVCAM-1 levels are also associated with an increased risk of death in type 2 diabetes [31]. In T1D, we found that in OVE26 mice, VCAM-1 and ICAM-1 increased to varying degrees after 24 weeks. As with other age-related chronic diseases, diabetes may be caused in part by the convergence of basic aging mechanisms underlying age-related tissue dysfunction, which include chronic “infertility” (independent of pathogens) inflammation, macromolecule damage, progenitor dysfunction, and cellular senescence [32]. Cell senescence is an essentially irreversible growth arrest that occurs in response to various cellular stressors, such as telomere erosion, DNA damage, oxidative stress, or carcinogenic activation, and is considered an antitumor mechanism [15]. Although senescent cells cannot divide, they are metabolically active. This high metabolic activity supports the release of proinflammatory cytokines, chemokines, and growth factors collectively known as SASP [33, 34]. In our study, we found that FVB mice had no abnormal increase in aging indicators at 24 weeks and 36 weeks. In OVE26 mice, both males and females had significant changes, but female mice had more changes than male mice such as p21 change. Whether these differences are related to hormone levels needs further experimental confirmation. Similarly, our study found that both SASP and related proteins were significantly increased. In view of the important role of SASP in aging, there are other SASP protein and related pathway changes that require further research. The association between diabetes and aging can be complex and complementary. On the one hand, the diabetic microenvironment may promote the development and accumulation of senescent cells, but on the other hand, senescent cells may cause tissue dysfunction and comorbidities observed in diabetes.

In conclusion, we demonstrate that T1D progression does cause senescence and can lead to fibrosis, ROS, and inflammation. However, the progression pattern and extent of these complications are different in males and females. This would be useful for choosing the OVE26 mice of appropriate age and gender in the future research of diabetic cardiomyopathy. Further investigations are requisite for elucidating the underlying mechanisms for the gender difference and may provide new therapeutic perspectives for the treatment of diabetic cardiomyopathy.

## Figures and Tables

**Table 1:** Echocardiographic analysis of male and female OVE26 and FVB mice. IVS; D, LVID; D, LVPW; D, IVS; S, LVID; S, LVPW; S, LV Vol; D, LV Vol; S, EF, FS, LV Mass, LV Mass corrected. *N* ≥ 6 in each group ∗ vs. sex- and age-matched FVB, *P* < 0.05; ∗∗ vs. sex- and age-matched FVB, *P* < 0.01; ^#^ vs. male, *P* < 0.05, ^##^ vs. male, *P* < 0.01.

Age	Sex	Genotype	IVS; d (mm)	LVID; d (mm)	LVPW; d (mm)	IVS; s (mm)	LVID; s (mm)	LVPW; s (mm)
4 weeks old	Male	FVB	0.55 ± 0.03	3.21 ± 0.29	0.6 ± 0.03	0.8 ± 0.07	1.77 ± 0.21	0.84 ± 0.09
		OVE26	0.55 ± 0.06	3.19 ± 0.32	0.59 ± 0.04	0.79 ± 0.09	1.71 ± 0.25	0.84 ± 0.1
	Female	FVB	0.56 ± 0.04	3.39 ± 0.17	0.65 ± 0.08	0.86 ± 0.05	1.86 ± 0.14	0.97 ± 0.07##
		OVE26	0.55 ± 0.04	3.19 ± 0.25∗	0.62 ± 0.08	0.83 ± 0.08	1.73 ± 0.19	0.88 ± 0.09∗

12 weeks old	Male	FVB	0.62 ± 0.02	3.77 ± 0.31	0.68 ± 0.03	0.86 ± 0.07	2.19 ± 0.23	0.97 ± 0.11
		OVE26	0.61 ± 0.02	3.88 ± 0.25	0.7 ± 0.05	0.86 ± 0.07	2.25 ± 0.19	1.02 ± 0.1
	Female	FVB	0.61 ± 0.03	3.64 ± 0.2	0.68 ± 0.04	0.86 ± 0.07	2.05 ± 0.16	0.97 ± 0.1
		OVE26	0.62 ± 0.03	3.73 ± 0.24	0.72 ± 0.05	0.89 ± 0.06∗	2.12 ± 0.17	1.01 ± 0.1

24 weeks old	Male	FVB	0.6 ± 0.02	4 ± 0.15	0.71 ± 0.03	0.89 ± 0.05	2.46 ± 0.15	1.04 ± 0.08
		OVE26	0.62 ± 0.02	4.04 ± 0.19	0.71 ± 0.03	0.86 ± 0.03∗	2.69 ± 0.16∗∗	1 ± 0.07
	Female	FVB	0.61 ± 0.02	3.94 ± 0.23	0.72 ± 0.03	0.86 ± 0.06	2.4 ± 0.16	1.08 ± 0.06
		OVE26	0.6 ± 0.02	4.01 ± 0.13	0.73 ± 0.04	0.88 ± 0.04	2.55 ± 0.11∗#	1 ± 0.05∗∗

36 weeks old	Male	FVB	0.63 ± 0.02	4.09 ± 0.13	0.72 ± 0.03	0.92 ± 0.03	2.52 ± 0.11	1.05 ± 0.08
		OVE26	0.6 ± 0.01∗	4.13 ± 0.22	0.7 ± 0.03	0.85 ± 0.04∗∗	2.79 ± 0.17∗∗	1.03 ± 0.06
	Female	FVB	0.61 ± 0.04	3.9 ± 0.16#	0.72 ± 0.05	0.87 ± 0.06	2.36 ± 0.12#	1.04 ± 0.07
		OVE26	0.6 ± 0.01	4.09 ± 0.09∗	0.72 ± 0.03	0.83 ± 0.04	2.74 ± 0.12∗∗	1.01 ± 0.03

Age	Sex	Genotype	LV Vol; d (ul)	LV Vol; s (ul)	% EF	% FS	LV mass (mg)	LV mass corrected (mg)

4 weeks old	Male	FVB	41.94 ± 8.87	9.57 ± 2.63	77.43 ± 3.31	44.98 ± 3.11	52.69 ± 8.62	42.15 ± 6.89
		OVE26	41.22 ± 9.55	8.85 ± 3.01	79 ± 4.27	46.6 ± 4.17	51.84 ± 9.97	41.48 ± 7.98
	Female	FVB	47.27 ± 5.45	10.67 ± 2	77.46 ± 2.97	45.18 ± 2.88	61.6 ± 7.17	49.28 ± 5.73#
		OVE26	41.18 ± 7.86	9.01 ± 2.72	78.42 ± 3.01	45.92 ± 2.81	53.41 ± 10.33	42.73 ± 8.27∗

12 weeks old	Male	FVB	61.2 ± 11.85	16.33 ± 4.2	73.62 ± 2.5	41.92 ± 2.05	81.26 ± 12.75	65.01 ± 10.2
		OVE26	65.33 ± 9.46	17.41 ± 3.51	73.55 ± 2.3	41.95 ± 1.91	85.76 ± 10.98	68.61 ± 8.79
	Female	FVB	56.14 ± 7.45	13.76 ± 2.77	75.63 ± 2.53	43.63 ± 2.2	75.79 ± 10.17	60.63 ± 8.13
		OVE26	59.69 ± 8.83	14.9 ± 2.97	75.18 ± 2.05	43.28 ± 1.78	83.08 ± 11.26	66.46 ± 9.01

24 weeks old	Male	FVB	70 ± 6.38	21.5 ± 3.2	69.41 ± 2.26	38.56 ± 1.74	90.9 ± 7.71	72.72 ± 6.17
		OVE26	71.72 ± 7.81	26.82 ± 3.72∗∗	62.7 ± 2.36∗∗	33.47 ± 1.65∗∗	93.14 ± 8.41	74.51 ± 6.72
	Female	FVB	67.99 ± 9.22	20.41 ± 3.21	70.06 ± 1.89	39.04 ± 1.49	90.47 ± 10.96	72.38 ± 8.77
		OVE26	70.48 ± 5.27	23.61 ± 2.52∗∗#	66.54 ± 2.12∗∗##	36.31 ± 1.61∗∗##	92.87 ± 7.06	74.29 ± 5.65

36 weeks old	Male	FVB	73.89 ± 5.28	22.83 ± 2.34	69.17 ± 1.37	38.42 ± 1.05	97.38 ± 6.93	77.91 ± 5.55
		OVE26	76.06 ± 9.26	29.52 ± 4.14∗∗	61.22 ± 2.25∗∗	32.48 ± 1.61∗∗	95.6 ± 7.82	76.48 ± 6.26
	Female	FVB	66.16 ± 6.35#	19.35 ± 2.34#	70.76 ± 2.1	39.59 ± 1.73	88.67 ± 12.53#	70.94 ± 10.03#
		OVE26	74.01 ± 3.83∗	28.17 ± 3.2∗∗	61.99 ± 3.12∗∗	33.03 ± 2.23∗∗	94.5 ± 2.73	75.6 ± 2.18

## Data Availability

The data used to support the findings of this study are available from the corresponding author upon request.
